# The Self-Simulation Hypothesis Interpretation of Quantum Mechanics

**DOI:** 10.3390/e22020247

**Published:** 2020-02-21

**Authors:** Klee Irwin, Marcelo Amaral, David Chester

**Affiliations:** Quantum Gravity Research, Los Angeles, CA 90290, USA; marcelo@quantumgravityresearch.org (M.A.); davidc@quantumgravityresearch.org (D.C.)

**Keywords:** simulation hypothesis, philosophy of mind, quantum mechanics

## Abstract

We modify the *simulation hypothesis* to a *self-simulation hypothesis*, where the physical universe, as a strange loop, is a mental self-simulation that might exist as one of a broad class of possible code theoretic quantum gravity models of reality obeying the *principle of efficient language* axiom. This leads to ontological interpretations about quantum mechanics. We also discuss some implications of the self-simulation hypothesis such as an informational arrow of time.

## 1. Introduction and Background

The simulation hypothesis [1] is a materialistic view, which argues that our universe is most likely a simulation in a physical universe. In *Are you living in a computer simulation?*, Nick Bostrom discusses how sufficient evolution of future technology leads to lifeforms capable of producing a large quantity of high fidelity simulations, called ancestor simulations. These simulations express an evolutionary process leading to humans and on up through higher levels of biological and technological evolution. The simulation hypothesis explains where the information that is our reality comes from. However, it does not offer an explanation for where the physical stuff of the real universe comes from. Because there would be more simulations than the one real universe, the deduction is that it is more likely that we are in one of the simulations than the real universe.

We discuss a non-materialist view called the self-simulation hypothesis, wherein everything is information, which we define as thought. The universe self-actualizes itself into existence via self-simulation using a mathematical code and a simulation *game rule* called the principle of efficient language. The salient idea is “timeless emergentism”, wherein the total simulation run can be viewed as one grand thought. Herein, the presumption of time does not exist, and, instead, a nested hierarchical order in the total self-simulation thought as an ordered set exists. Emergentism in this context is where the self-simulation grand thought has various sub-thoughts in a nested hierarchy that synergistically composite to higher-order sub-thoughts and eventually composite to become the grand thought of the self-simulation run itself. An early sub-thought in the ordered set is the base mathematics of the self-simulation code. Another important early sub-thought is the principle of efficient language, which is the idea of economizing sub-thoughts, called code-steps or actions, for the purpose of economically expressing the meaning that emergent sub-thoughts, such as humans, choose to experience, such as a measurement. One important sub-thought early in the ordering of the nested hierarchy is a fundamental particle.

*Materialism* is an axiom that says matter and other physical things, such as light and spacetime, are the fundamental substances of reality. In this view, the terms “abstract” and “physical” are juxtaposed, where abstract, i.e., not real, information merely describes the real physical things. The axiom offers no explanation for where the physical stuff comes from. It just is.

Digital physics [2,3,4,5,6,7,8,9,10,11] is a non-materialistic view, which says there is no physicality but only abstract information as the fundamental building blocks of emergent informational structures of reality, such as atoms. One advocate was John Wheeler, who discussed how entities capable of observing and thinking influence how physical information behaves. The digital physics view is non-dualistic in that it does not juxtapose the terms “abstract” and “physical”, since everything real is information and therefore abstract. However, just as the materialist view does not explain where the physical stuff comes from, the digital physics view does not offer an explanation for where the information comes from. It just is.

Idealistic holistic panpsychism [12] (herein, panpsychism) is related to ontological idealism [13]. It says that everything is thought or consciousness. Panpsychism philosophy does not explain how the panpsychic substrate comes to exist. It just is. We propose a panpsychic self-simulation model, which attempts to describe the origin of the panconsciousness.

In Reference [14,15], one of the authors discussed the idea of the self–simulation. (After release of our preprint, Christopher Langan contacted us to point out that some of our independently derived ideas are similar to some of his. Upon review of his work [16,17,18], we acknowledge some similarities, such as the use of the term “self–simulation” [17]. Where “simulation” is defined as something representing something else, a self-simulation is a case where something simulates, i.e., represents or creates itself. The universe creating itself is a rather ancient idea. To our knowledge, the modern idea of reality as a code–in–action was first introduced in 1969 by Finkelstein in *The Space–Time Code* [19]. In essence, the work of both Langan and ourselves is a synthesis of three general ideas: (1) reality is made of information in the form of code/language [2,3,19,20,21,22,23], (2) reality is a transtemporal system, where things from one time can influence things in other times [24,25,26] and (3), the substrate of this information is panconsciousness [12] that emerges from itself, as the creator or simulator of itself. Differences in our synthesis include our connection to self-referential vs non-self-referential symbols, strange loops, a finite possibility space for physical information, a unified free energy principle, quasicrystalline codes and details on the nature of retrocausality, to name a few). In 2017, he put forth the idea that a digital physics–based quantum gravity theory can be based on simplex–integers or shape as number [27]. A unification of the numerical aspect of the digital physics view and geometric aspect of the materialistic view was proposed, where the shapes that form reality are equivalent to numbers. In Reference [15], he discussed how these shape–numbers as physical information can act as the elements of a quasicrystalline mathematical code. This view provides an origin for the panpsychic substrate of reality, where nothing “just is” and where there is a logically self–consistent origin story for the panpsychic substrate as a self–simulation strange loop. The logic is based on evidence that consciousness can evolve to increase in magnitude, as it has done here on Earth from early lifeforms to humans. Human minds can run primitive simulations, such as dreams. Sufficiently large minds can be networks of smaller minds and can, in principle, evolve to enormous levels in the future to run fully realistic universal self–simulations. A panpsychic self–simulation unifies notions from emergentism and panpsychism by showing that panconsciousness can emerge from or self–simulate itself.

One of the goals of this paper is to stimulate debate by providing critical thinking options for those interested in comparing the simulation hypothesis to the self-simulation hypothesis. We have evidence today that computers are getting more powerful, along with the resolution of the physical systems they can simulate. We also have evidence that self-simulations, i.e., dreams of one’s physical form, have gotten extremely powerful as consciousness has evolved here on Earth. If it were a competition for resolution and detail, human dreams are today more realistic than computer simulations. Some have had lucid dreams, wherein one realizes they are dreaming. What is most remarkable is the ultra-high-fidelity resolution of these mind-based simulations and the accuracy of the physics therein. Upon investigating the quality of these simulations, it is typical for lucid dreamers to report that these mind-based simulations are generally indistinguishable from ordinary reality. In fact, most people who dream are usually not aware they are dreaming because the simulation is so realistic that one tends not to question it. The reader might take their experience now, as they read these words, and notice that they have not suspected they are in a dream at this moment. Why would they? The experience has a very high resolution—but so does a dream. The physics of dreams can be impeccable insofar as how light triangulates off glasses of water, how gravity behaves, etc. More powerful minds can simulate more fine-grained physics, perhaps down to the Planck scale, as the self-simulation hypothesis predicts. We are not saying that human dreams are a physical reality, however, since the physical reality is the dream of the panconsciousness, not a human.

In the simulation hypothesis, as opposed to the self-simulation hypothesis, the first consciousness emerges out of a physical realm. This then leads to those consciousnesses creating technology that leads to ancestor simulations, where more consciousness emerges from the pure information of the simulation. If we presume that the probability of our reality being an ancestor simulation is likely, this same logic implies that each ancestor simulation is likely to be inside another ancestor simulation. While the self-simulation hypothesis can have mental simulations within mental simulations, all simulations are made of the same stuff—thought. Specifically, one might question, in a simulation hypothesis ontology, if they are physical, i.e., real, or if they are merely information in one of the nested simulations within simulations. However, because the self-simulation hypothesis is based on panpsychism, where everything is thought, it does not require a physical universe with computers to run the mental self-simulation. The difference here is that physical information can emerge out of a conscious realm rather than just physical information emerging from a physical realm. Accordingly, the question “Which is the real universe?” is resolved because all information that is thought of is real and there is nothing outside of thought or consciousness. In the simulation hypothesis, the simulations are a fake reality. In the self-simulation hypothesis, they are real.

As mentioned, panpsychism does not explain the origin of the fundamental thing—the panconsciousness itself. It just is. Materialism does not explain the origin of the fundamental thing—the physical stuff itself. It just is. Digital physics does not explain the origin of the fundamental thing—the information itself. It also just is. Similarly, the simulation hypothesis is based on materialism. Thus, it does not explain the material stuff. It just is.

The self-simulation hypothesis explains the origin of the fundamental thing—the panconsciousness—and does not say, “It just is”. The universal mind self-actualizes itself into existence via the strange loop of self-simulation. As mentioned, this requires the assumption that reality, i.e., the panconsciousness, is outside of time. Notions of quantum gravity, such as the Wheeler–de Witt equation and Rovelli’s insights, suggest that time is not real [28,29]. Furthermore, as opposed to the ontology of digital physics, the self-simulation view offers an explanation for where the information that is reality comes from, including the information of the panpsychic computational substrate itself. The computational mental substrate emerges from its own self-simulation as thought. The idea is that the physical simulation of spacetime and particles is mathematical thought, which emerges from the evolution of mind in the simulation in a logically consistent loop or whole. Loops or circles do not necessarily mean that an argument is faulty circular reasoning. The self-simulation hypothesis is a strange loop, a term introduced by Hofstadter, who claimed that the self is an inherently circular structure with no grounding [30,31]. Strange loops that describe creation are paradoxical if one assumes linear time. We can ask: “How did the panconsciousness come to exist?” The creationist who thinks panconsciousness is fundamental with no origin story, i.e., “Panconsciousness just is”, will say that is a false question. The self-simulation hypothesis ontology would answer, “The panconsciousness self-actualizes itself in a strange loop via self-simulation.”

The only price to pay is that one must reject the false question: “Which came first, the chicken or the egg?” or “what came first in time, the simple math of the self-simulation code or the complex emergent panconscious substrate that thinks of the simple math?” We move away from classical ideas of time and causality. This suggests that the universal mind self-actualized itself through a grand thought of a complex system (a thought that is itself the universal mind) that has evolutionary hierarchical nested complexity that provides explanation for each level in the hierarchy as emerging logically from all the others.

We claim above that materialism, panpsychism, and information theoretic ontologies do not explain the origin of the fundamental stuff and that the self-simulation hypothesis does. Those three ontologies do not try to explain the origin of the fundamental things. Similar to most religions, all three are creationist insofar as saying there is one fundamental thing that creates all others but which itself has no creator. The self-simulation hypothesis recognizes that: (1) thoughts called choices of what to think; and (2) thoughts called the experience of those chosen thoughts are fundamental and that one explains the other via the self-simulation creative process of self-actualization. Materialism does not say that fundamental physical stuff created itself. Digital physics does not say fundamental information created itself. Panpsychism typically does not say that the universal consciousness created itself. They say that fundamental things “just are” with no explanation. In this paper, we build a logical thesis that does not contradict the parts of these other three ontologies that claim the fundamentality of “physical = materialism”, “information = digital physics”, and “consciousness = panpsychism”. For us, all three things, physicality, information, and consciousness, are fundamental. We show how they are equivalent and discuss a categorization and origin scheme that simply goes further into the process of relating and explaining than those more limited ontologies that stop at “it just is”. Instead, using code theory, we provide theoretical structure suggesting how reality self-simulates. We provide various forms of evidence-based rationale.

As discussed, panpsychism is the idea that all of reality exists within a mental substrate, where everything is thought. Physicists, such as Roger Penrose, discuss versions of panpsychism in relation to quantum mechanics [32]. Shan Gao [33,34] discusses how conscious beings can distinguish definite perceptions and their quantum superpositions, while systems without consciousness cannot distinguish such non-orthogonal quantum states.

Interpreting quantum mechanics (QM) places one at the nexus where the philosophy of what is real (ontology), experimental physics and mathematics converge. More physicists subscribe to the Copenhagen interpretation than any other, which requires entities capable of observation to collapse wavefunctions. While some interpret Copenhagen to not refer to consciousness, this obscures the role of consciousness in measurement, since it is typical to recognize that observers must be conscious or are defined with consciousness. Derivative off-shoots, such as Wigner and Stapp’s *consciousness causes collapse* interpretation [35,36] and Qbism [37,38], also postulate that consciousness is the fundamental quantum operator or actualizer of reality. If we assume that we possess consciousness and freewill and follow the many-worlds interpretation, it is conceivable to imagine that freewill leads to decoherence, similar to outcomes of measurement. Consider an electron that is spin up in the z-direction. We flip a coin; heads leads to a spin measurement in the y-direction, while tails leads to a spin measurement in the x-direction. This would lead to four universes of equal probability: spin up y, spin down y, spin up x, and spin down x. The random coin flip can be replaced with a conscious choice, suggesting that all freewill choices could be combined as a mixed state within the many-worlds interpretation, such that a conscious choice is a type of decoherence (One objection may be that it is simpler to create an infinite ensemble of coin flips, rather than an infinite ensemble of conscious freewill choices, but we do not look to model probabilities associated with freewill). Not all interpretations of many-worlds must lead to this, but this is one possibility. For clarity, we interpret freewill choices as a form of wavefunction collapse, but note that a self-consistent many-worlds interpretation may also be valid.

Practically minded physicists often minimize the scientific importance of dealing with the philosophical meanings of what QM is trying to tell us. If they are not trying to push the bounds of fundamental physics, such as developing a quantum gravity theory, then it is true they can, in the words of David Mermin, “Shut up and calculate!” [39]. However, for those of us looking to understand nature more deeply or working on quantum gravity theories, we cannot shut up. We must critically think about consciousness and certain aspects of philosophy that are uncomfortable subjects to some scientists. When physicists trivialize those working on such crucial issues, it helps limit the probability of advancements in fundamental physics. Accordingly, we share a few encouraging remarks from titans of modern physics about the importance of this study.

**Erwin Schrödinger**: *Consciousness cannot be accounted for in physical terms. For consciousness is absolutely fundamental*.

[40]

**Arthur Eddington**: *The stuff of the world is mind stuff*.

[41]

**J. B. S. Haldane**: *We do not find obvious evidence of life or mind in so-called inert matter...; but if the scientific point of view is correct, we shall ultimately find them, at least in rudimentary form, all through the universe*.

[42]

**Julian Huxley**: *Mind or something of the nature as mind must exist throughout the entire universe. This is, I believe, the truth*.

[43]

**Freeman Dyson**: *[M]ind is already inherent in every electron, and the processes of human consciousness differ only in degree and not in kind from the processes of choice between quantum states which we call “chance” when they are made by electrons*.

[44]

**David Bohm**: *It is implied that, in some sense, a rudimentary consciousness is present even at the level of particle physics*.

[45]

**Werner Heisenberg**: *Was [is] it utterly absurd to seek behind the ordering structures of this world a “consciousness” whose “intentions” were these very structures*?

[46]

**Andrei Linde**: *Will it not turn out, with the further development of science, that the study of the universe and the study of consciousness will be inseparably linked, and that ultimate progress in the one will be impossible without progress in the other*?

[47]

**John Bell**: *What is much more likely is that the new way of seeing things will involve an imaginative leap that will astonish us*.

[48]

**Frank Wilczek**: *The relevant literature [on the meaning of quantum theory] is famously contentious and obscure. I believe it will remain so until someone constructs, within the formalism of quantum mechanics, an “observer”; that is, a model entity whose states correspond to a recognizable caricature of conscious awareness*.

[49]

Other notable authors interpreting that physical reality requires consciousness to choose to do measurements in order to actualize states of reality include von Neumann [50], London and Bauer [51], Wigner [52], Stapp [53,54], and Penrose and Hameroff [55,56,57,58,59].

### Definitions

Because discussions of consciousness are philosophical, it is necessary to establish the following definitions for key terms used in this document.

**Strange Loop**: A hierarchical structure that is wrapped back upon itself, where the simplest object is embedded in the whole or the most complex emergent part and where all parts depend upon all others and where the emergent whole is dependant upon the synergy of parts.

**Thought**: The process and result of choosing or creating meaning, where “meaning” is always a relationship.

**Meaning**: A relationship between two or more objects recognized or created by an entity capable of doing so. For example, if we think of the compound self-referential symbol of a square, we may recognize the relationship between two vertices as a meaning. However, if we think of the symbol of a heart, we may create, via relationship, the symbolic meaning of love, as opposed to recognizing some inherent self-referential meaning implied in the symbol of the heart. An entity capable of deciding that this is like that or this is like this is an entity capable of generating meaning, i.e., thought. Meaning can be recognition of inherent relationships or the creation or assignment of arbitrary ones.

**Object/Symbol**: Defined as in set theory, an object is anything that can be thought of. All objects are symbols because all thoughts are symbolic, i.e., have meaning.

**Symbolism**: A case where an entity capable of perceiving meaning relates or equates one object with another object—a relationship within thought, where X = anything; either itself or something else.

**Self-referential Symbolism**: A special case of symbolism, where an object represents or relates to itself; “this means this” or X = X. For example, one can use a square to self-referentially symbolize a square or its body diagonal to represent the number $\sqrt{2}$ or the quantity of vertices of a square to represent the quantity 4.

**Non-self-referential Symbolism**: A special case of symbolism, where an object represents or relates to something other than itself; “this means that” or X=anything other than X.

**Mind/Conscious Entity/Consciousness**: A system capable of choosing to perceive and create meaning. For example, if one were to feed all thoughts or meaning into your mind, at the expense of you being able to choose your own thoughts, you would be a clone or mirror of their consciousness. According to our definition, the discretion to choose what to be aware of or what to think cannot be disassociated from the term consciousness.

**Information**: Symbolic meaning, which implies thought. Put differently, all thoughts can be reduced to symbolism in the mind of an entity capable of actualizing information. While this is a different notion than quantum information, our mathematical program (beyond the scope of this paper) is to find code theoretic simulations that lead to the emergence of quantum information.

**Emergent Physical Thought (EP)**: Information as self-referential symbolic thought (number and shape) that forms the basis of physical reality; spacetime and particles.

**Emergent Consciousness Thought (EC)**: Information as non-self-referential symbolic thought, such as freewill decision to observe, the sense of self, humor, etc. that forms the basis of consciousness reality emergent from physical reality in the strange loop.

**Code/Language**: A finite set of symbols with relational or ordering rules that include syntactical freedom. Codes may be used by entities capable of strategically exercising syntactical choices for the purpose of expressing meaning.

**Freewill/Choice**: A non-random and non-deterministic action or state that ontologically exists and that is a member of a set containing at least one other such possibility that does not ontologically exist because it has not been actioned, recognized, observed, thought, chosen, or any other suitable term that separates the subset from the super-set. Freewill or choice may be significantly influenced by other things/thoughts but not fully controlled. In order for the choice to be non-random and non-deterministic, there must be reason, strategy, whim, theory, or some other process of thought. Put differently, if the action occurs due to thoughts, it is by definition non-random and non-deterministic. It is sometimes suggested that freewill is an illusion and that everything is deterministic or that everything is a combination of determinism or randomness. While possible, it is far-fetched when one considers, for example, the idea that an Emily Dickson poem was a result of such a deterministic process combined with randomness, an accidental process. While reductio ad absurdum proofs are not strong, they can be instructive for choosing the lesser of evils when no good proof is available.

**Measure/Observe**: The choosing of symbolic meaning. For example, you observe an experiment and creatively generate, i.e., choose, meaning in your mind about the experience, which includes influence or interaction. The choice may be conscious or subconscious.

Interestingly, all 14 of the above terms are forms of “thought” in our ontology, wherein everything is thought.

## 2. The Self-Simulation Hypothesis

### 2.1. Axioms and Principle of Efficient Language

The self-simulation hypothesis (SSH) allows for an application of the *principle of efficient language* (PEL) [15]. The SSH is built upon the following axioms:

1. Reality, as a strange loop, is a code-based self-simulation in the mind of a panpsychic universal consciousness that emerges from itself via the information of code-based mathematical thought or self-referential symbolism plus emergent non-self-referential thought. Accordingly, reality is made of information called thought.
2. Non-local spacetime and particles are secondary or emergent from this code, which is itself a pre-spacetime thought within a self-emergent mind.
3. The panconsciousness has freewill to choose the code and make syntactical choices. Emergent lower levels of consciousness also make choices through observation that influence the code syntax choices of the panconsciousness.
4. The desire or decision of the panconscious reality is to generate as much meaning or information as possible for a minimal number of primitive thoughts, i.e., syntactical choices, which are mathematical operations at the pre-spacetime code level.

These four axioms can be briefly summarized by the notions of (1) the strange loop of the emergent self-simulation that includes (2) emergent spacetime, (3) emergent freewill sub-entities and (4) the PEL. The last axiom leads to an energetic model, where the behavioral statistics of the code are based on a least computational principle and where those statistics can change over the evolution of the self-simulation—depending upon what choosers decide what information/meaning the PEL should economized for. Due to this economy principle, the code used will be a member of the set of maximally economical codes capable of generating the physical reality we observe, such as a universe with particles possessing the spin and charge values shown in experiments. Here, “economy” is defined as the amount of consciousness-based resource used for the fundamental mathematical actions (thoughts) in the code for the purpose of expressing some chosen meaning, such as a particle’s pattern of propagation through space and internal time. Accordingly, the SSH, which subsumes the PEL, is applicable to any code-theoretic quantum gravity theory that is a member of the most economical set of codes simulating reality.

The SSH is non-deterministic and yet posits that there are hidden variables or a sub-quantum mechanics that also define a quantum gravity theory, where spacetime and particles are patterns therein.

To work in an efficient manner, the panconsciousness breaks down all freewill choices into a binary decision tree, as this is the most efficient way to to express possibilities. This leads to a trivalent graph network. Quantum gravity as an effective quantum field theory has been riddled with infinity problems due to infinite graviton vertex diagrams. However, recent advances in scattering amplitude computations find that trivalent (cubic) graph representations lead to more efficient computations [60,61,62]. Similarly, Wolfram conjectures that reality is a cellular automata built upon an ideal trivalent graph code [9]. The *quasicrystalline spin network* [63,64,65,66], used in our program at Quantum Gravity Research, is a trivalent graph. A corollary of the PEL is, therefore, that the panconsciousness chooses a trivalent graph network because it is maximally economical for simulating quantum spacetime.

### 2.2. Information

Before elucidating the SSH, some pre-discussion about information, i.e., symbolism, is called for. The Copenhagen-like interpretations divide things into two ontological categories—unreal information and real physical “stuff”—which is an epistemological view. For example, the probability distribution of the wavefunction is considered unreal because it is merely information, while a measurement updates the wavefunction to a state more closely resembling the post-measurement physical reality and also actualizes some physical reality into existence—that part correlated to an observation. In other words, it defines realism as physical stuff and something unreal as information or abstractness. However, another popular view, digital physics, is that all of reality is made of information or abstractness, which Wheeler described as “it from bit”. Since reality is real, they say information is real. Materialism is monism. Copenhagen is, in some sense, dualist because, unlike materialism, it places abstract information at a fundamental status in the form of the wavefunction, and thus it has the dualism of information and physical stuff playing two juxtaposed fundamental roles.

The SSH is monistic in that it views reality as information defined as thought. If the SSH were to suppose reality uses a wavefunction, it would say that both it and physicality are made of the same stuff—thought. It relies on the PEL, which posits that there are two fundamental forms of thought or symbolic information:

1. Self-referential symbolism that is part of a mathematical spacetime and particle code based on the thought of number and geometric symbols or pixels of spacetime information (the case of X=X)
2. Non-self-referential symbolism (X=anything other than X, such as the thought of humor, love or a decision of purpose to observe/measure a physical system)

Unlike the Copenhagen interpretation and other ontologies assuming physical stuff to be the opposite of information or thought, the code theoretic ontology of the SSH traffics only in information or thought. Ontology is the study of what is real and what is not, so it is binary. The binary ontology of the materialism-based Copenhagen view is to say that information is unreal and physicality is real. We invert part of this. We set up our binary ontological system to be (See also [67]):

1. The unreal potential information as thought that could exist.
2. The information actualized by thought (by observers including the panconsciousness substrate) selected from the possibility space of nonexistent potential information.

For example, imagine thinking a thought that is so strange and complex you can assure yourself no thinker in the universe previously thought it. Before you thought of it, it was not actualized information. However, it was information that could have existed if you thought of it earlier. Accordingly, we have potential self-referential information that could exist if thought of. Furthermore, we have physical states that could exist as physical thought made of self-referential symbolism along with states that have been thought of by the universe through our observations and so do exist because thought of. We also have thoughts as a form of information that can influence measurements (e.g., decisions to measure) and, in so doing, influence the physical information via wavefunction collapse or something akin to it.

As discussed in [15,27] and following our definitions, symbols are objects of thought that represent themselves or something else. As mentioned, any symbol use fits into one of these two categories: X=X and X=anything other than X. The X=X category is that of self-referential symbolism. This symbolic meaning is special because it possesses non-arbitrary or non-subjective truth. For example, via mathematical first principles, the numerical properties of a triangle, such as its area, may be deduced non-subjectively from the symbol itself. One may use an equilateral triangle to represent the meaning of itself—an equilateral triangle. In this case, the meaning of the symbol is not subjective. Alternatively, subjective meaning can be chosen for the triangle, such as the notion of change symbolized in physics by the triangular delta symbol. Quasicrystals can be created by projectively transforming self-referential symbols called Lie algebraic root lattices. Some of these lattices encode gauge symmetry physics via their associated Lie algebras. Our program of code theoretic based physics, derived from quasicrystalline root lattice transformations, is the X=X case, i.e., self-referential symbolism. Unlike lattices or crystals, quasicrystals are self-referential symbolic codes, where their syntax rules are non-invented, i.e., are implied by mathematical first principles.

Languages, i.e., codes, are systems that have an irreducible class of symbol types called “letters” and syntactical rules. Users can steer the syntax degrees of freedom in choices of how to order of the symbols to create semantic form, i.e., meaning, that can exist in nested hierarchies of emergent symbolic meaning, including spatiotemporal or geometric meaning. Letters can be combined to form the emergent meaning of “words”. Words can be combined to form the meaning of “sentences” and so on. One can recognize this as synergistic meaning, where the emergent meaning is greater than the sum of the irreducible symbols or letters and where no additional base symbols are needed for the extra synergistic meaning—only the strategic ordering of the symbols.

Our approach is to build a physical ontology based on a finite set of self-referential geometric symbols that map to formal algebras. We call this level of thought or information *i*, where the lower case represents the idea that it is base or letter-level information. Because it is a discrete spacetime code, our pixels of self-referential symbolism are shapes such as quasicrystalline prototiles or, alternatively, entire quasicrystal *inflation* [63,68] states. These geometric objects map to various isomorphisms and bijections in the form of mathematics that are not geometric. For a finite quasicrystal possibility space, there is a finite set of N different inflations that can be performed. This results in a superset of N! possible ordered sets or dynamic patterns that can be generated from N. Quasicrystals are proper codes, which require freewill choices of syntactical degrees of freedom or code action to form meaningful expressions. Codes or languages do not organize themselves. They require action—the decision of a syntactical chooser—for the addition of each new symbol in a sequence. This is in contrast to crystals, which are not codes and where the positioning of one tile determines all others. With quasicrystals, “fundamental” particles may emerge as phason quasiparticles that can be created as ordered sets of inflations, wherein the order may be chosen by the panconsciousness as it gets “instructions” from its sub-part consciousnesses, e.g., humans, called observations/measurements.

How would such “instructions” from a human observer to the panconsciousness syntactical chooser occur? Clearly, we are suggesting mathematical actions that the panconsciousness operates that relate to the Planck scale, but humans are at the meter scale. We are not sure of the mechanism. On the one hand, we may presume that since the panconsciousness is a great mind and we are subminds of it, it knows our thoughts of observation as its own sub-thoughts. This should be true. However, perhaps it is also true that there is a deep mathematical connection between the panconsciousness substrate and our EC thoughts to measure and think other things. After all, our thoughts emerge from Planck scale EP information and up through higher-order spatiotemporal EP physical symbolism, such as DNA and biological structure in a fully connected continuum. Penrose theorizes that there is an ideal Platonic substrate at the Planck quantum gravity scale that interacts through structures in our body called microtubules. He believes that there is a panconsciousness at the Planck scale that interacts with us through these structures in our body near the angstrom scale, according to he and Hammeroff’s *orchestrated objective reduction* (Orch-OR) theory [55]. This is related to our quantum gravity program because of two similarities. The first is that our quasicrystalline mathematical substrate is built of 3D tiles based on the five Platonic solids, which we derive via rigorous means from the transformation of certain Lie algebraic root lattices. These structures include Fibonacci sequence numbers and various rational and irrational numbers useful for gauge symmetry physics. Accordingly, our mathematical formalism is built upon an ideal Platonic substrate. The second similarity is that microtubule structures encode Fibonacci numbers and are better described technically as quasicrystalline, not crystalline atomic motifs. Quasicrystalline mathematics, materials quasicrystal science, and the very term quasicrystal are arcane with fewer than 100 physicists and mathematicians funded to work full time in these areas. Microtubules behave as a binary code as implied by sequences of coherent patterns of charge sign value changes to their dimer substructures.

Paola Zizzi extended the Orch-OR framework into cosmology, using a quantum computational paradigm, showing how the universe became conscious at the end of the inflationary period [69]. Her view is different from ours in terms of how we use evolutionary biology, where we see evidence that consciousness has emerged in at least humans. We take this forward and assume that, just as simple lifeforms, as cells, self-organize collectively to exhibit emergent human consciousness, lifeforms such as humans can self-organize to exhibit collective emergent super-consciousness that is far greater than the sum of the parts. One can think of a human mind–body system as a percentage of spacetime and energy in the universe that self-organized to exhibit emergent consciousness. In principle, all spacetime and energy in the universe can self-organize to form an uber emergent consciousness. Accordingly, it seems that our process of explanation through evolutionary hierarchical processes may be able to converge mathematically with aspects of Penrose and Zizzi’s views insofar as all of spacetime becoming a quantum net capable of thought. As opposed to our view, where, from our vantage point, panconsciousness emerges forward of us in the hierarchy we call “time”, Zizzi’s view is related to a universal consciousness emergence event in our “past” at the end of the inflationary period driven by “dark energy”.

We believe that, until a predictive quantum gravity theory is discovered, it is premature to speculate on the nature of dark energy and matter. What we appreciate about Zizzi’s idea is that reality itself can be a quantum net capable of quantum computation. In general, neither Penrose and Hammeroff nor Zizzi focus on topological quantum computation but instead on standard quantum computation. At low temperature, atomic quasicrystals are topological phases of matter. Mathematically, our Planck scale based quantum gravity program is based on topological quantum computing. Criticisms by Tegmark and others about Penrose’s Orch-OR mathematics are often centered on the contention that microtubules in the human body cannot quantum compute to describe consciousness because the body is not at a low enough temperature. The high temperature leads, in these objections, to decoherence times that are too short for the Orch-OR model to make sense. Penrose and Hameroff have certain arguments to rebut this. However, one way to resolve it is to switch from the notion of quantum computing to topological quantum computing, which allows local thermally induced decoherence events without the destruction of the global quantum superposition state—the global qubit.

With respect to the collection of all ordered sets within the superset N! mentioned above, it is a statistical possibility space with probabilities governed by an energetic scheme we use based on a least computation principle built into the PEL. As mentioned, our interpretation of QM is general and can be applied to other spacetime codes or code theoretic quantum gravity models. However, we use this quasicrystalline interpretation of reality as an illustrative example of the PEL, since our interpretation of QM is related to a class of quantum gravity models and axioms such as ours.

Computer simulations endowed with a random number generator to represent the non-determined choices to measure can implement a form of a *game of life*. A random number generator may select actions from a deterministic unitary evolution that weighs the selection probabilities of different orderings, i.e., phason quasiparticle random walks, via their computational economy for expressing paths of extremal action. The fundamental particles, as patterns emerging from self-referential geometric symbols, such as 3-simplices, are themselves emergent self-referential spatiotemporal symbols built of simpler symbols that reduce to on/off states of 0-simplices (points) in the possibility pointset that we build our graph actions on. This discrete point set and the internal structure of the quasiparticles are made of such geometric self-referential symbolism, which encode both fundamental and emergent numerical values. Similarly, higher-order emergent spatiotemporal patterns emerging from these, such as “atoms” and “molecules”, are also self-referential symbols or what we call *emergent physical symbols*, but where our physicalism is information/thought-theoretic. Because the possibility space is discrete, the degrees of freedom are finite, i.e., the random walks such quasiparticles can take are of a finite quantity of possibilities. These emergent strata of higher-order physical symbolism above the level of *i* are ranked in terms of compound complexity.

Tononi et al. have a similar idea for a ranked complexity measure, where, at some critical magnitude, the complexity can be defined as consciousness or thinking. His approach is called *integrated information theory* (IIT) [70,71,72]. IIT posits that many physical systems intrinsically possess consciousness, which, in this context, is defined as a measure of a subsystem’s ability to affect the rest of the system, i.e., causal reality [72]. His notion of consciousness and freewill is comparable to ours in the sense that a highly conscious entity would be able to make more freewill choices, which increases its ability to influence the syntactical degrees of freedom in the code. In this sense, the emergent consciousness of the universe—the simulation substrate—would be the most conscious entity. However, subsystems of this conscious mind may emerge within the self-simulation and possess consciousness once such subsystems become complex enough to create meaning, i.e., observe or think. Such perceived meaning of a subsystem is also a perceived meaning of the panconscious substrate and so is a form of distributed workload of choice actions to think, i.e., generate meaning/information. This connectivity of our consciousness to the substrate instructs the panconsciousness to make fine grained mathematical code choices that comport with our more coarse-grained thoughts called observations. In other words, there are a large number of different choices of mathematical action at the quantum gravity level that the panconsciousness can choose from in order to map to a given approximate experience of an observation that a human or other thinking entity does, thus we provide: (1) the instructions for when the panconsciousness does a mathematical choice/thought/action; and (2) a coarse-grained constraint on what those choices can be in order to equate with the meaning we thought of in the form of the observation itself.

Our approach is different from IIT insofar as using language theory, where we see a nested hierarchy of code-theoretic information referred to as $EP_{1}$, $EP_{2}$, $EP_{3}$, ⋯, $EP_{N}$, with EP standing for *emergent physical* information. Since EP or physical information is geometric and numeric, and therefore non-subjective, all forms of higher-order EP are emerging from base-level self-referential symbols. However, not all self-referential symbols need to be EP, since we can think in our minds of EP objects such as triangles without it existing as physical information. The different levels represent different strata of complexity emerging from simpler strata in the same sense that a molecule is emerging from atoms, which are emerging from fundamental particles, which may be emerging from self-referential geometric symbolic Planck units of spacetime information/thought. To account for all information, we must sum the total amount of *i*, which, in our case, is the quantity of on/off state selections in the quasicrystal inflation possibility point space, with the sum of total information in the emergent hierarchy of $EP_{N}$. Physicists are generally trained to think only in terms of base or $EP_{1}$ information, such as spin states. Accordingly, an analogy would be helpful to emphasize the informational power of higher-order or emergent information.

Consider a book with *N* letters and randomize them. If we have a value of 1 unit for the meaning or information of each letter, the magnitude of i=N units. We notate the total information of the system as *I*. In this case of random ordering of *i*, we have that i=I. However, if we allow the letters to be organized into words, we have more information than *N*. It is not easy to agree on the magnitudes of the emergent information. However, at the same time, it cannot be ignored because the emergent information in a complex physical system is statistically causal on all parts of the system in a force-like manner, as with the notion of *entropic force* [73] or Tononi’s notion of complex system information influencing the behavior of the system. For example, we may randomize the letters in a string of DNA code, which leaves only the information of the sum of the molecular letters. However, if we allow them to be ordered in a meaningful way, they encode a protein folding algorithm, which is an immense statistically causal package of information influencing lower entropy systems that surround it that must be credited with some unknown value of causal information that is over and above the sum of the letters. Accordingly, we have

(1) $$I=i+EP_{1}+EP_{2}+\cdots +EP_{N}.$$

In this notation, $EP_{j+1}$ denotes the additional emergent information in relation to the synergistic meaning created from combining multiple elements of $EP_{j}$.

At some point in the nested EP hierarchy of this emergent physical information, something enigmatic occurs that may be related to something very similar to the Orch-OR model. Consciousness emerges from the regime of EP to form a new system of information that is itself unbounded in possibilities over a finite set of EP based *i*. Let us call this regime EC for *emergent consciousness*-based information, i.e., non-self-referential thought. As mentioned, all non-self-referential symbols are forms of EC, but EC may also include non-physical self-referential symbols, such as the thought of a square. We propose that EC also exists in a nested stratification of $EC_{1},EC_{2},EC_{3},\cdots ,EC_{M}$, each built upon previous strata in the hierarchical stack. This regime is capable of generating additional information over and above the emergent physical EP information. We argue that the potential of its possibilities is infinite, as it is the universe of all things which can be thought of. Since all forms of emergent information must fit in one of these meta-categories, EP or EC, we have that

(2) $$I=i+EP_{1}+EP_{2}+\cdots +EP_{N}+EC_{1}+EC_{2}+\cdots +EC_{M},$$

for the total system information *I*. Again, the EP physical information is self-referential geometric and numerical symbolism, such as spacetime quanta with quasiparticle patterns representing fundamental particles with their quantum numbers, spacetime quanta geometric numerical values, and the statistical numerical values relating to the economy rule of the PEL.

To appreciate the scientific importance of the EC enigma, we reference some unresolved problems and some experiments. First, there is no consensus on the definition of “consciousness”. Thus, herein, we mean the definition we provided in the definitions section, which reduces down to thought or meaning. One might think issues of consciousness are solely the purview of psychologists and philosophers, as opposed to physicists. A second problem is the measurement problem, which relates to the difficulty of reconciling the completeness of the wavefunction, linear evolution, and the Born rule with respect to measurements. If consciousness relates to measurement, then consciousness is relevant for quantum mechanics. The third problem is the question of how consciousness emerges from things that are not conscious. This is called the *hard problem of consciousness* [74,75]. The SSH starts with the notion that consciousness is fundamental and self-emerges as a strange loop in a cosmological holism ontology that requires abandoning the idea that time is fundamental or even real.

The SSH is novel in how we use the EP information of numerical and geometric mathematical thought to create high-order compound EP physical thought that evolves to EC thought eventually capable of self-actualizing itself in one grand thought that is itself the strange loop i→EP→EC→i→EP→EC→⋯, where *i*, EP, and EC are part of the overall self-simulation thought. However, unlike other attempts to resolve the last two problems above by saying panconsciousness is the ground of reality without explaining how it emerges or why consciousness would influence physical things, such as collapsing wavefunctions, our approach goes further by providing an origin explanation for panconsciousness that, similar to humans, can think in both the EP and EC regimes.

The empirical evidence is that physical systems change when conscious minds choose to observe them. This is reminiscent of how a video game player with a VR headset has a relationship with the code processing computer, where she instructs the CPU and GPU to compute and render simulation landscapes according to what she observes. For the most part, it is the physical evidence for and the inexplicability of observers changing physical reality which leads to all of the interpretations of QM, such as the Copenhagen version. These interpretations, whether they call it “observer” or “consciousness”, often place measurement in the role of collapsing wavefunctions (or decoherence), as evidenced, for example, by the change to the interference pattern in a double slit experiment. Simply having knowledge of which slit a photon goes through dramatically changes the physical system.

At early stages of 20th century physics, there was more debate about whether it was the conscious knowledge of the observer or some physical interaction within the experimental apparatus, such as in the detector, that caused the physical changes in the interference pattern. However, as experimental physics and discussion advanced, it became more widely agreed that it is consciousness, i.e., knowledge or thought about the measurement that generates the physical change and not a physical interaction between an artificial or biological detector and system being observed. Our model implies that, if a consciousness were to somehow be able to have awareness of a physical system without using ordinary means, such as photons or sound, that it would collapse wavefunctions without need of any form of physical detector. Radin et al. reported evidence of this, showing a 4.4 sigma deviation above the null effect [76,77,78]. Tremblay independently analyzed the results to confirm the statistical significance but also identified lesser magnitude statistical anomalies in the control data [79]. The implication of our model would caution that even the control data might be contaminated by EC-thought based human influence. This is because the entire experiment should be permeated by opinions and thought about the meaning of the endeavor, even the control aspect but with a less focused or less potent degree of statistical modulation from the baseline statistics of QM.

Semantic confusion can enter these discussions. For example, we used certain words above emphasizing the term “consciousness”. Other authors use terms such as “measure” and “observe”. However, these terms are inextricably linked with words such as “awareness”, “knowledge”, “consciousness”, and “thought”. For some Copenhagen-like interpretations of quantum mechanics, at some point of demarcation in a self-organizing system, such as a human, consciousness emerges, which is capable of collapsing the quantum wavefunction and changing physical systems via awareness or knowledge from and about observations [53,80]. This seemingly mystical phase transition is often referred to as the *Heisenberg cut*. For us, the thinking needs to be able to create EC information, which requires an emergent mind capable of abstraction. We believe the most sophisticated and plausible mechanism to date for the Heisenberg cut is the Penrose and Hameroff view.

In conclusion of this section, the hierarchical stratification of our EP and EC information does not allow for a limit on the magnitude of total *I* that a system can have because the EC information possibility space is unbounded. The SSH resolves the measurement problem by showing that codes use choosers of syntactical freedom. Choices themselves are thoughts, thus there are choices being made by emergent entities, such as humans, that generate EP and EC. Stephen Hawking asked: “What is it that breathes fire into the equations and makes a universe for them to describe?” The SSH posits that it is observers that animate the syntax expressions of the code, which then map to the statistical equations of a post-quantum mechanical quantum gravity formalism that includes a set of gauge symmetry equations. In this sense, the foundational thought is the observation choice thought/action, which maps to mathematical choices corresponding to quantum gravity code syntax choices, that is the irreducible building-block thoughtform of the universe. When such choices are made to observe and think about physical systems, this generated thought informs the panconsciousness of the meaning created by observation, which defines the degrees of freedom for mathematical choices it can make at the spacetime code level.

The SSH resolves the hard problem more weakly than the Orch-OR model by recognizing that consciousness, i.e., information or thought, is the only thing that exists but without disagreeing with the basic premise of Orch-OR. However, it does this in a very different manner than typical idealist panconsciousness approaches, which say “Consciousness just is”. The SSH offers an explanation for how consciousness comes to exist via self-emergence through the logical strange loop of the i→EP→EC→i→EP→EC→⋯ simulation, where we do not need to say that consciousness “just is”. There is an origin story. The panconsciousness requires the thought of physical mathematical symbolism to self-emerge from. This is because only through simple-to-complex information or language theoretic structure can the free bonus information or synergy of emergent information exist, where the whole is greater than the sum of the parts. In the universe of all thoughts, mathematical information may be the simplest of all. For example, an object is a thought, and the simplest object is either the empty set or dimensionless point, depending upon how one argues it. Thus, the grand self-simulation thought is a nested hierarchy of thoughts, mostly of the synergistic emergent form, starting with the dimensionless point, in a state of on, off, or undecided, and ending with the thought of the entire self-simulation thought, which is equal to the panconsciousness substrate itself.

### 2.3. Nonlocality of the Self-Simulation

At its deepest level, we interpret the measurement problem as being related to questions of how consciousness can perform measurements. It should be noted that decoherence is not the same as wavefunction collapse. Quantum systems in nature can decohere without measurement because non-measurement based decoherences exist in the unitary evolution of off equilibrium systems that are not perfectly isolated. In some systems near a tipping point of decoherence, a measurement can change the physical system enough to cause it to decohere. It is difficult to imagine having a completely satisfying explanation for the measurement problem without a consensus definition of consciousness. We know that consciousness is thought and awareness and the other terms in the definition section that are equivalent to thought. However, we still cannot deeply explain consciousness other than our experience of it and recognition of its synonyms.

The *simulation hypothesis* proposes that reality could be a computer simulation, which implies it is made of code [1]. Physicists Beane, Davoudi, and Savage propose this can be experimentally constrained [81]. Simulations typically run with a finite number of resources. This may be accomplished by discretization of spacetime, as in several quantum gravity theories, such as ours (emergence theory). One of the proposed experimental signatures to look for would be an anisotropy in the distribution of cosmic rays that is consistent with the simulation hypothesis. Campbell et al. have proposed experimental tests for the simulation hypothesis [82]. These same tests could be applied to the SSH if it turns out these experiments suggest that we are in a simulation.

Again, the simulation hypothesis is based on the dualistic idea that there is a physical reality and various non-physical or information theoretic simulated realities. The SSH rejects this dualism and suggests that it is more probable that we are in a mental self-simulated universe, which may be less far-fetched than the idea that we are in a simulation living in a different physical universe. One reason is Occam’s razor and another is evidence. Specifically, in [14,15,27], one of the authors discusses a cosmology that allows for a code-theoretic universe to self-simulate or self-actualize itself into existence from the “future”. A similar cosmology was discussed by [17]. Consciousness that emerges at late stages of the self-simulation eventually evolves to a magnitude sufficient to hold, abstractly in pure informational thought space, the quantum gravity code necessary for its own self-simulation run or self-evolution starting at the big bang. This strange loop is similar to a mind running a simulation from an initial condition and where that simulation becomes the mind itself after a long run-time. Of course, the self-simulation idea requires time to be an emergent illusion, as discussed by Rovelli [29] and in different terms by Susskind and Maldacena [83].

Advanced waves have been interpreted as being related to consciousness or measurement in multiple QM interpretations. Aharonov was inspired by Feynman to create an independent advanced wavefunction, leading to the two-state vector formalism [25,84]. This is also compatible with the transactional, many worlds and Bohmian interpretations. Sutherland has generalized Bohmian mechanics, which leads to a notion of post-quantum mechanics and introduces new nonlocal dynamics [85,86]. The evidence for non-locality is sufficient enough to presume spacetime, whether fundamental or not, is non-local [87,88,89,90].

Accordingly, we may adopt a physical logic: A influences B influences C influences A influences B, and so on. That is, an emergent mind-like substrate of the universe in the future can self-actualize itself by creating the code and initial conditions to run its own simulation—all within the abstraction of pure self-simulated information in the form of choice, observation or awareness as a strange loop with hierarchical order but not time. We may think of this with time and presume it to be non-local or presume time to be illusionary, emergent or non-fundamental. The substrate, then, is made of information called thought or consciousness. Its evolutionary self-simulation run is also made of the same abstract information, i.e., *meaning* within thought or mind-stuff [12,32,33,34,36,91,92,93]. Sarfatti’s interpretation of Sutherland’s extension of Bohmian mechanics [94] suggests that the wavefunction is itself a property of consciousness, while the particle is the domain of material reality. In this dualistic view, dividing reality between physical stuff and consciousness, advanced waves allow for consciousness to have new retrocausal dynamics on the physical stuff. From this perspective, consciousness of the future can influence the past so long as freewill is not violated.

Our view is similar to Sarfatti’s interpretation, except that we do not take a Bohmian approach and divide things into physical stuff and consciousness. It is all information in the self-actualized strange loop of panconsciousness, where the physical information is EP thought and the non-physical information is EC thought. Our approach is also different in that we subscribe to a discretized spacetime view. Bohmian mechanics does not.

It is noteworthy that if the large scale causal structure of the universe is a strange loop, instead of having some beginning for which we have no explanation, then perhaps there would be empirical implications, which may ultimately be observed.

With this ontological background in place, let us discuss the regime of EC information. When we think of the idea of love or politics, is this self-referential symbolism? Is it information? Of course, these are not cases of self-referential symbolism. However, they are indeed two networks of compound information. These packages are not physical information, such as the self-referential geometric information of the base-code elements in the simulation that can be organized into emergent levels of self-referential EP, e.g., a biosphere or solar system. However, EC is equally as real and causal as EP information and so must be considered part of the universe’s total information along with i and the regime of EP. Each conserved stratum of EP information is built upon the conserved strata under it. To be more precise, the choices of EP configuration are conserved over a finite simulation run time because the degrees of freedom in a discrete possibility space-based code, such as ours, are finite. There are a finite number of possible animations or ordered sets of on/off state selections on the finite quasicrystal possibility space. The quantum states therefore exist in a finite-dimensional space.

This is not the case for the possibility space of EC information that can be created over a finite simulation run. The EC category is the regime of non-self-referential symbolism, where we can say X=anything other than X, and where both *X* and *anything other than* *X* are members of the set *anything which can be thought of*. Put simply, we may think about anything from an infinite set of things to create in our mind. Again, in an information only universe, this form of EC information—thoughts—is equally as real and influential as the self-referential physical information that the universal consciousness self-simulation substrate can hold. Thus, the regime of EP possibilities is a set of selections or relationships in a finite possibility space. It is discrete and may lead to a quantum gravity formalism with a discretized probability density distribution. The regime of EC possibilities is the infinite set of all possible symbolic relationships and combinations thereof, i.e., meaning that one can choose to think of from the infinite universe of possible thoughts. Unlike the EP space, the EC possibility space is smooth and continuous—infinite. Sequential choices in the regime of thought or consciousness of what and when to measure create reality by coding a concatenation network of wavefunction collapses—forming a completely different universe than the highest probability path(s) through the unitary evolution if no measurements had occurred. It creates a network of short-lived unitary evolutions between measurements defined by the freewill choices of conscious entities to strategically order sequences of measurements. A measurement itself is a thought in the mind of the observer, which prematurely kills off or terminates the deterministic unitary evolution that existed prior to the measurement and after the last measurement by creating a new function that will live until the next observation and thought about it. All functions exist in the possibilities of the Hilbert space. The choices of when, where, and what to measure are EC thoughts that can be chosen to change the unitary evolution of the EP information.

**Figure 1:**
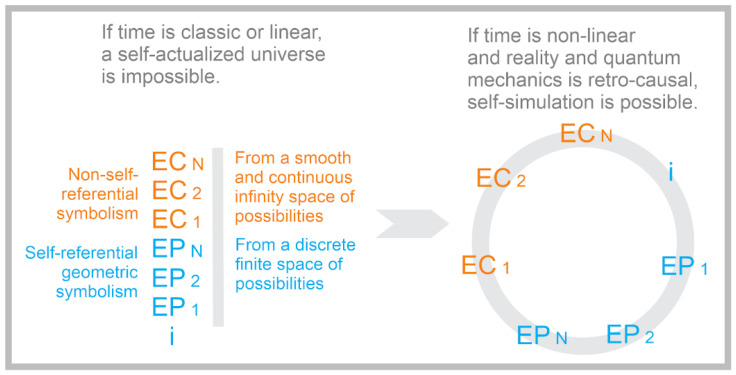
Self-Simulated Universe: Humans are near the point of demarcation, where EC or thinking matter emerges into the choice-sphere of the infinite set of possibilities of thought, $EC_{\infty }$. Beyond the human level, physics allows for larger and more powerful networks that are also conscious. At some stage of the simulation run, a conscious EC system emerges that is capable of acting as the substrate for the primitive spacetime code, its initial conditions, as mathematical thought, and simulation run, as a thought, to self-actualize itself. Linear time would not permit this logic, but non-linear time does. Furthermore, rejecting the conjecture of time and seeing it as an illusionary aspect of our perception permits it. This model may be more scientifically satisfying than the materialist statement, “Mass, energy and spacetime *just are* without explanation”. Here, we may ask: “Where does the mind-like universal substrate of self-simulation thought come from?” We get a scientifically satisfying answer or at least a logically consistent one. It emerges, just as human consciousness did from simpler thoughts. Even without knowing exactly how, we need not accept that it just magically appeared. We can ask: “Where did the Planck scale information theoretic EP building blocks come from and where do they live?” In our view, they are information in a simulation run in the mind of the emergent panconscious universe—the self-emergent substrate as a strange loop.

As shown in Figure 1, conscious humans are part physical information (made of the numerical and geometric spatiotemporal information/thought in the EP regime) and part abstract non-self-referential symbolic thought from the EC regime. The commonalities between the EP and EC regimes include the following:

1. They are both mutually dependent upon one another for their origin and existence. They each interact with one another.
2. They are both made of pure symbolic information in a language-theoretic informational paradigm, where relationships between two or more symbols of meaning at one stratum form synergistic meaning, and therefore higher-order symbols of meaning that form a new higher-order symbolic stratum.

In order for the SSH to be causally consistent, EC must emerge to be complex enough to allow for a self-simulation. This is what allows for the completion of the causal circle or emergent whole. If EC were to never emerge, the system would never gain any consciousness, and the SSH would be illogical. Relativistic time as we know it is irrelevant in relation to the panconsciousness because the self-simulation strange loop is created as a whole or grand thought, which, from a wavefunction perspective, includes advanced and retarded waves. Since the self-simulation requires emergent consciousness to run in the first place, this makes the likelihood of emergent life to be inevitable. Driven by the PEL, the panconsciousness can send advanced waves that encourage emergent structures, such that additional observers can emerge.

Of course, there are systems in the $EP_{1}$ regime of fundamental particles, such as a rock, that do not contain meaningful EC information. There can also be emergent consciousnesses in the EC regime that, unlike humans, are not partially in EP and EC. For example, coherent patterns of pure EC information can allow the emergence of higher-order collective consciousnesses that is not at the level of the panconsciousness but that is, in some sense, a higher level than human animal-level consciousness. Furthermore, there can be coherent patterns of mathematical information, such as topological information, at the quantum gravity regime that can be understood as consciousness but which is not made of fundamental particles. There may be topological consciousnesses that can emerge that are trans-temporal in nature, which, of course, would be an entirely different form of thought than we are familiar with. However, the EP and EC regimes cannot be disassociated, since the hierarchical stack of information based symbolic systems is interactive, non-locally connected and self-embedded.

As mentioned, this model posits that our consciousness instructs the panconsciousness to project the wavefunction to a new state upon measurement. Our thesis does not imply that the panconsciousness must always follow what we currently label as the laws of physics or QM. If this were true, it would be something that can be tested for using humans who envision things that violate physics or influence the statistics of QM [76,77,78]. Because emergent conscious entities, such as us, instruct the panconsciousness substrate to perform mathematical operations in the EP regime that comport with our EC thoughts about our surroundings or things non-local to us, such violations of canonical physical theories appear to be allowed.

Other than the consciousness activated collapse of the wavefunction, there is another way to recognize how EC or consciousness is physically causal. The following thought experiment is instructive. Consider a system of particles that is organized as a conscious human at the bottom of a deep energy well, such as the base of a mountain. The human may create an abstract story in her mind that climbing out of the energy well every day is a good and strategic thing to do. Only because of that abstract belief and choice, living in pure EC, the probability for all the particles of her body to do this each day will be close to 100%. Quantum statistics would never predict this because it is not the statistics of the behavior of conscious systems. Consciousness, via observation, is what kills off the deterministic unitary evolution of the wavefunction in a manner not described by the Schrodinger equation. QM merely describes statistical fluctuations that lead to emergent classical conservation laws. QM discusses statistical dynamics between measurements. Consciousness determines a process of sequential measurements via self-reflection and external observation.

On the other hand, we can imagine a woman at the bottom of the hill being devoid of consciousness, such as with a brain-dead state but with all the particles in her body in the same states as when she was not brain-dead. In this case, devoid of consciousness, the probability of her climbing a macroscopic hill approaches zero and reproduces the statistics of classical mechanics, as she does not have enough consciousness to influence causal reality via free will to expel energy. That is, the evolution of her system would follow the unitary evolution of a wavefunction, where the probability to go up the hill would comport with QM and be very close to zero.

It is trivially true that freewill or the conscious ability to choose to go against the statistics of classical and quantum physics is a fact relating to some unknown foundation beyond QM. Consciousness causes a deviation from the statistics as a type of non-ergodic causal entropic force. For example, the desire for a human to experience novelty may affect the statistics to prefer a less likely path and motivate her to climb a mountain. This claim is also supported by the fact that the canonical interpretations of QM hold a special place for consciousness insofar as it uniquely being capable of actualizing reality into existence from a non-real space of quantum possibilities into the space of physical realism.

## 3. Informational Arrow of Time Based on Measurement

One can think, at time-1, of the idea of a person leaving their home. At time-2, they can think of the person moving from there to a store. These are two thoughts. However, if one did not accept the idea of time, they can think of a single thought as an ordered set of elements in pure information or thought space. The total information of the ordered set is a single thought or object without need for time but relying on order or pattern to express the information formerly labeled as “time”. For convenience of language, we use terms such as “first”, “then”, “before”, “after”, and so on. Here, those terms mean the notion of “order within a set”, where the set is the self-simulation thought of the universe. For example, one can watch a movie and have the laser reading head of their DVD player relate to each bit on the disc one moment at a time in an ordered set. Conversely, one may look at all the bits in one moment with no notion of a flow of time. In either case, there is order in the set of bits.

Keeping in mind how we use “first” above in the thought of the person leaving their home, the SSH stipulates that the “first” measurement, i.e., thought, is by the emergent panconsciousness substrate. It chooses the thought of the mathematical code, quasiparticle construction, a function corresponding to the initial variables, and other mathematical thought necessary for the total thought of the self-simulation “before” the measuring sub-thinkers such as us take the load of choice actions in a form of distributed computing or distributed thinking.

To simplify, let us focus on the idea of a function. By way of metaphor, consider the Mandelbrot set fractal and a quadratic map that could create it with enough computational resources. The fractal and the quadratic map are different objects. The second object, as a primitive algorithm, is a much simpler thought than the first. That is, more symbolism and mathematical operations are necessary to create the first object than the second. One possesses more information and takes more thought than the other. There is a class of functions that describe objects such as series. Some of these functions allow one to access the *n*th element of a series without knowing the n−1 preceding elements. One example is the Binet formula fb(n) that allows one to know the *n*th Fibonacci number without knowing any of the earlier ones,

(3) $$fb\left(n\right)=\frac{{\left(1+\sqrt{5}\right)}^{n}-{\left(1-\sqrt{5}\right)}^{n}}{2^{n}\sqrt{5}}.$$

The unitary evolution of the wavefunction gives, for example, the probabilities for measuring a particle’s position at time-2, time-3, and so on. We may take the position coordinates for each time and order them by probability magnitude in a column. We can do the same for time-2, time-3, and so on to create a table. Each row of the table would be a series of complex values. The key here is that, according to QM, the series has order and is not random. For example, it is known that the spectral lines of the hydrogen atom have quasiperiodic order. Our emergence theory quantum gravity formalism has probability tables such as this. However, the statistics emerge from the Fibonacci number-based underlying mathematics that we derive via the transformation of Lie algebraic root lattices. We are currently exploring functions and algorithms that will allow us to “dial in”to one column in the table without having to calculate all the other columns. Herein, let us call this function $F_{n}$.

Let us return to the idea of the initial thought about the starting mathematics of the simulation. Let us call this $F_{n}$, where the function is not the same object as the table or information of the unitary evolution. It is exceedingly simpler thought/information. Based on the initial conditions as variables to the function that define it as being $F_{n}$ and not $F_{m}$, we have at some evolution of the non-computed function a high probability for a measuring or computing entity to emerge, just as we have in the ordinary quantum formalism with a single universal wavefunction expressing a deterministic unitary evolution from the big bang to the first computing or measuring entity.

Early in the series corresponding to this function, the quality of order in the unreal possibility space described a universe too hot for life to form. That is, the probabilities for a measuring/computing entity—a thinker—were very low. Somewhere in the unitary evolution, the first measuring life statistically appeared at the *N*th column of the aforementioned table with a high probability for existing. The first measurement occurred; the first moment of conscious awareness in the super-thought of self-simulation as a strange loop. However, we must be careful here to think about what this means with our EP and EC labeling scheme. What was created, EP or EC, or both? What was the nature of the EP information? Measuring entities create coarse grained EP and more fine-grained EC information. For example, when we do a position measurement, we are creating numbers and geometry. We elaborate. First let us return to the previous idea of self-referential symbolism. We used examples in geometry, such as a triangle representing a triangle. However, numbers are also an example. There is self-referential non-subjective truth in the idea of a square’s body diagonal being $\sqrt{2}$ of its edge length. There is also non-subjective truth in the number 5 being prime or the factorial set combinatorics of the number 120. As mentioned, our previous work elaborated on the notion of self-referential numerical symbols called simplex-integers.

Again, when we do a position measurement, we are doing very coarse-grained measurements of numbers and geometry. Presuming Planck time and volume pixelization of spacetime, our position measurement creates some of the EP information in the form of number and geometry that is a spread of positions in a geometric coordinate space, since we do not have the ability at this stage to do position measurements with Planck scale resolution. We are also spreading this over the geometry of ordered sets of spatial selections that we call “time” because we do not have time based resolution finer than the attosecond level. This same measurement “blur” we create as real geometric EP information is associated with pure numerical EP numbers, as probabilities, attached to each of the various geometric coordinate values in the blur that we call a position measurement. Put differently, our notion of a position measurement is really a statistical spread in a region of spacetime that is far smaller than our assumptions of where the particle might be prior to the second measurement based upon the prior measurement that we extrapolate from. In addition to the EP information, the observer may generate some non-self-referential information or story about the experience of measurement, such as “beauty” or an idea related to some physical theory the observer holds in their mind.

When we consider the aforementioned table of rows and columns, we can understand that a column correlates to a “random walk” for a particle, where the column itself is not in a single Planck moment. It is a short animation that we can call the random walk and the column has the probabilities for all such random walk animations at that coarse-grained level of time we are measuring at. Each box on the column is a brief animation or ordered set that describes a given minimal random walk and its statistical probabilities, which map to how many computational actions are needed for that minimal walk. A walk of distance X may take more or less computational actions than another walk of distance X. Let us say that the quantity of boxes on the column prior to the measurement is N. Next, we have the measurement. As explained, the EP level measurement information that is created is not precise down to the Planck level. Accordingly, it is smeared out in a blur of uncertainty. However, it is not as smeared out as the probability distribution prior to the measurement. The measurement can be interpreted in this framework as lensing or concentrating the probabilities into a much smaller set of squares in the column, such that most of the previous squares now have zero probability values and where the remaining squares have much higher probabilities than if no measurement occurred to define the approximate time and space resolution of information created by the observer.

According to the PEL, the panconsciousness substrate would have to have a reason for computing or simulating a precise position and time value down to the Planck level. The SSH view is that the panconsciousness, as an emergent consciousness, leaves the heavy lifting of new thoughts to the conscious measuring entities within it. That is, its emergent consciousness grows on the substrate of the network of all conscious thoughts of EP and EC within it. However, after this first course grained measuring entity does this very first measurement, additional measuring entities emerge. Today, we have 7.7 billion human measuring entities on Earth and it should be true that many other living things on this planet, at least, can also measure to create EP information, even if they are not generating as much EC information as humans are. We can see now that we have an informational “arrow of time” or pattern in the ordered set. More measuring entities create more total information. In addition, the quality of measurement can increase. A human has a conscious moment of awareness about once every $10^{41}$ Planck moments. However, in principle, this frequency can greatly increase with evolution, especially with artificial evolution via technologies such as CRISPR gene editing. We can see here a picture in our table, where the density of contracted or probability lensed columns is increasing, as the quantity of measuring entities increases. We can also see that the resolution toward the Planck scale of space and time can improve, which increases the degree to which a column is probability lensed. The limit of contraction for a given column is measurement at the Planck scale of time and space that lenses the probabilities onto a single box, leaving the other boxes with zeros. The evolutionary nature of the SSH implies that the magnitude of EP and EC information increases exponentially as one goes deeper into the ordered set of the self-simulation super-thought of the universe. It has appeared enigmatic to some as to why there seems to be a sense of directionality or flow to the experience we call “time”, considering the equations of canonical physics are generally time reversible. This evolutionary view of increasing information is certainly one aspect of empirical observation. We see a universe starting with a quark gluon plasma with low degrees of complex information in the EP regime to a more complex universe of hydrogen atoms to a universe with over 100 elements to solar systems, biospheres, DNA, and up through massive amounts of EC information being generated by mankind at this stage. This observed directionality of complexity is the informational arrow of time, where the term information is not restricted to quantum information, i.e., $EP_{1}$.

One form of this complexity is measuring entities. Some readers may wonder what happens in this self-simulation picture when measuring entities become so numerous and can measure at the Planck scale, such that all columns are collapsed to single boxes. Would the simulation end because no further choices can be made via measurement? Would that imply a “game reset” reset for another simulation run? Of course, if the answer is “yes” to both, the SSH would imply a cyclic cosmology model. For now, we would answer this question with the response, “Perhaps”. Our model is at a mid-level stage of development, and our focus at this time is on reproducing quantum statistics from first principles and deriving gauge symmetry equations for a new quantum gravity and particle physics model.

### A Free Energy Principle from the PEL

The emergence of biological life and its ability to preserve nonequilibrium states has puzzled many. Schrodinger introduced a term called negative entropy [95], which was later shortened to negentropy. Later, Schrodinger explained how he was actually referring to free energy. Entropy maximization corresponds to free energy minimization. While there is a notion of thermodynamic entropy and thermodynamic free energy, it has been realized for roughly a century that the total entropy includes information entropy, suggesting the same holds true for free energy. Friston introduced a free energy principle in the field of neuroscience as a mechanism to have complex biological systems preserve a nonequilibrium thermodynamical state [96]. Friston’s free energy principle includes a free energy function of the internal state of a biological system that makes decisions of belief about its environment. It essentially states that life attempts to model reality and minimize the difference between its mental model and reality.

Considering our notion of EP and EC, it is plausible that the thermodynamic free energy principle and Friston’s free energy principle can be combined into a single free energy principle based on *I*. The emergence of intelligent life can be thought of as an equilibration of consciousness. Initially in the timeless strange loop, the panconsciousness is highly conscious, yet the emergent information is not very conscious. In other words, the panconsciousness is modeling itself by spreading its consciousness and free will nonlocally in a manner reminiscent of distributed computing or decision making. The emergence of EC and more intelligent life may allow for physical information to stay in non-equilibrium states because the emergence of additional consciousness is also part of the equation.

There are a few self-consistencies in assuming that EC relates to Friston’s free energy principle. EC has been related to the measurement problem. In order to talk about Friston’s principle, a complex biological system must model reality, which is a type of quantum measurement. Friston’s principle seems to implicitly motivate the evolution of consciousness, as more highly conscious entities and greater quantities of such entities would be able to more accurately model reality at ever finer resolutions relative to the *pixelation* at the Planck scale. Friston’s free energy principle has been connected to consciousness [97], although they do not adopt our nuanced panpsychic view. Connecting EC to Friston’s free energy principle may be helpful for the development of a mathematical formulation of consciousness in terms of variational Bayesian methods.

From the perspective of the panconsciousness as the largest source of EC, it would prefer to model itself with increasing accuracy. To do this efficiently, it helps to provide EP, which is more grounded and universal as a self-referential language. Rather than modeling the abstract realm of EC, it can learn more about itself by creating some primitive notion of EP. This process continues until EP emerges to a regime of complexity where it can form thoughts and model aspects of its reality. It may be conceivable to envision emergent EC via EP as an efficient code running on multiple parallel processors. Rather than the panconsciousness completely modeling itself, it can let the emergent information model reality and create fundamentally new interactions of physical information, which leads to a deeper understanding of the panconsciousness as a whole.

## 4. Unification of Emergentism and Panpsychism

It is widely believed that panpsychism is incompatible with emergentism [98]. We have defined panpsychism above. Emergentism is the belief in emergence, particularly as it involves consciousness and the philosophy of mind. Its antithesis is reductionism, because emergentism is the property where the emergent whole is greater than the sum of the properties of the system’s parts. Theories of consciousness generally fall under one of these two categories: Consciousness is present at a fundamental level of reality (non-self-simulation-based panpsychism) and has no explanation or origin or it emerges from simple physical processes that exist primordially with no explanation (non-self-simulation-based emergentism). As articulated, the transtemporal interactive nature of the SSH requires emergentism to explain the existence of the panpsychic computational thinking substrate of reality to run its own self-simulation. Accordingly the SSH resolves the conflict between emergentism and panpsychism—unifies the two views.

Emergentism typically subscribes to the philosophy of materialism. While our panpsychic view is opposite from materialism, our model nevertheless has the notion of emergent consciousness (EC) from emergent physicality (EP), both of which are forms of thought in the strange loop of the self-simulation. In this sense, our view is philosophically different than emergentism, yet effectively accomplishes the same goals, as a type of consciousness emerges from physical information, which emerges from the panconsciousness, and so forth.

## 5. Spiritualism

Whether one appreciates the term “spiritualism” or not, aspects of the SSH and our emergence theory program include aspects of spiritualism if we follow these definitional excerpts from *Encyclopedia Britannica.*

Spiritualism, in philosophy, a characteristic of any system of thought that affirms the existence of immaterial reality imperceptible to the senses. So defined, spiritualism embraces a vast array of highly diversified philosophical views.

Furthermore:

Less obviously, it includes belief in such ideas as finite cosmic forces or a universal mind, provided that they transcend the limits of gross Materialistic interpretation. Spiritualism as such says nothing about matter, the nature of a supreme being or a universal force, or the precise nature of spiritual reality itself.

The ideas laid out in this document as a whole cannot be defined as spiritualism because, as the above definition states, spiritualism says nothing about matter, the nature of the supreme being or a universal force, or the precise nature of spiritual reality itself. Our thesis does indeed say several things about those ideas, as we focus on issues such as mathematical physics, symbolism, and the finite but evolving nature of the supreme being and its origin story, i.e., the emergent panconsciousness substrate as a strange loop. We have not used the term “God” in place of panconsciousness in this document because that is an ambiguous and confusing term. It has many meanings. Two of the most general meanings associated with that word are ideas anathema to this thesis. The first is that God is infinite. This is not the case in the SSH model. Our panpsychic substrate evolves. The second is the popular idea in many religions that God creates everything. Our panpsychic self-simulation is everything and is collectively created by everything within it. It is unitary and interdependent, where the panconsciousness cannot exist without evolving through us and everything else that can make decisions.

Creationist ideas are non-unitary and non-interdependent. That is, the thing that creates everything is not itself created by those things. This is not just an idea of religion. It is general, as the idea that there is one thing fundamental, such as God or spacetime and energy or information. Furthermore, the one underlying fundamental thing does not require other things to exist or other things to create it. Those things may only emerge from or be created by the fundamental thing. Modern materialism is one such view, where spacetime and energy just are, and they evolve according to eternal primordial physical laws to create us and the rest of reality. All interpretations of QM mentioned above are creationist views in this same sense. Panpsychism and digital physics are also creationist views. The SSH is not a creationist view. It is the philosophy of holism, which is the idea that properties of a system are synergistic and can only be explained by the emergent whole and where the whole influences and creates the parts and vice versa—mutual co-creation of all.

When such a philosophy can fit within a logical or mathematical model of reality that makes predictions and explains things with reasoning, a scientifically minded person may choose to ask questions that are not “allowed” by today’s status quo scientific views. For example, one can ask whether or not such a new physics would support the possibility that a consciousness can exist across time and without having to be dependant on systems of atoms, as with biology. The SSH allows this. One might ask whether thoughts can interact in a manner that comports with ideas such as “communicating with a higher form of consciousness including one’s future evolution or even the panconsciousness substrate” or “communicating with another consciousness non-locally without requiring propagating signals in spacetime”. For both questions, the non-fundamental nature of time in the SSH allows this possibility.

## 6. Conclusions

We introduce the self-simulation hypothesis as a modification of the simulation hypothesis. We consider the assumption of mental simulations to be more plausible than computer simulations because, as with lucid dreams, they are currently more precise. Furthermore, humanity’s recent hacking of evolutionary biology, via CRISPR gene editing, is likely to allow rapid evolution of consciousness in the future—*designer consciousness*—that can make mind-simulations even more powerful. Future non-local quantum gravity theories and deeper understanding of what consciousness is may allow new forms of mind to emerge from networks of biological consciousnesses or that do not require matter in the first place. The upper limit of energy in the universe that can self-organize into conscious systems and networked systems of conscious systems is 100% of the energy.

As an overall takeaway message throughout the document, we also state that mental simulations are a better fit for what quantum mechanics and the measurement problem seem to be implying. Our overall theme that bridges ideas ordinarily at odds has been the notion of code theory or hierarchical inter-nested symbolic systems in a strange loop and how we recognize thought and the universe itself as such.

We contrast our holism viewpoint with the “it just is” viewpoints of panpsychism, materialism and digital physics and point out that it is more true to the scientific spirit of demanding origin stories for everything, even those things that are supposedly fundamental. We speak in limited terms of the program worked on at Quantum Gravity Research, called emergence theory. We also discuss various interpretational aspects of QM. We end with some controversial but hopefully stimulating ideas about spiritualism and the possible importance of humanity’s role.

## Abbreviations

The following abbreviations are used in this manuscript:

QM	Quantum Mechanics
EP	Emergent Physical Thought
EC	Emergent Consciousness Thought
SSH	Self-Simulation Hypothesis
PEL	Principle of Efficient Language
Orch-OR	Orchestrated Objective Reduction
IIT	Integrated Information Theory
CRISPR	Clustered Regularly Interspaced Short Palindromic Repeats
